# Emerging non-viral vectors for gene delivery

**DOI:** 10.1186/s12951-023-02044-5

**Published:** 2023-08-17

**Authors:** Chenfei Wang, Chaolan Pan, Haiyang Yong, Feifei Wang, Tao Bo, Yitong Zhao, Bin Ma, Wei He, Ming Li

**Affiliations:** 1 Department of Dermatology, Children’s Hospital of Fudan University, National Children’s Medical Center, Shanghai, 201102 China; 2 School of Chemical Engineering and Technology, Xi’an Jiaotong University, Xi’an, Shaanxi 710049 China; 3 Department of Anesthesiology, The Second Affiliated Hospital of Air Force Medical University, Xi’an, Shaanxi 710032 China; 4 School of Basic Medical Sciences, Fudan University, Shanghai, 200032 China; 5 School of Medicine, Anhui University of Science and Technology, Huainan, Anhui 232000 China

**Keywords:** Gene therapy, Gene delivery process, Non-viral gene vectors, Nanomaterials, Lipid nanoparticles

## Abstract

Gene therapy holds great promise for treating a multitude of inherited and acquired diseases by delivering functional genes, comprising DNA or RNA, into targeted cells or tissues to elicit manipulation of gene expression. However, the clinical implementation of gene therapy remains substantially impeded by the lack of safe and efficient gene delivery vehicles. This review comprehensively outlines the novel fastest-growing and efficient non-viral gene delivery vectors, which include liposomes and lipid nanoparticles (LNPs), highly branched poly(β-amino ester) (HPAE), single-chain cyclic polymer (SCKP), poly(amidoamine) (PAMAM) dendrimers, and polyethyleneimine (PEI). Particularly, we discuss the research progress, potential development directions, and remaining challenges. Additionally, we provide a comprehensive overview of the currently approved non-viral gene therapeutics, as well as ongoing clinical trials. With advances in biomedicine, molecular biology, materials science, non-viral gene vectors play an ever-expanding and noteworthy role in clinical gene therapy.

## Introduction

The development of COVID-19 vaccines has sparked widespread interest. mRNA-based therapies are rapidly gaining attention owing to their unique advantages in quickly developing vaccines and immunotherapy for various ailments [1, 2]. Given that most human diseases stem from genetic factors, gene therapy represents a promising modality for addressing various inherited or acquired disorders by replacing faulty genes or silencing genes [3]. Gene therapy encompasses the targeted exploitation of genetic material, which includes gene replacement through DNA or mRNA [4, 5]; gene silencing utilizing siRNA or miRNA [6], and CRISPR-Cas9 based gene editing [7].

However, achieving safe and efficient gene delivery to specific cells requires overcoming multiple biological barriers, including extracellular obstacles such as enzyme degradation, serum protein interactions, electrostatic repulsion of genes and cell membranes, and innate immune system, as well as intracellular obstacles such as endosomal escape, transport barriers, precise release [8]. Therefore, gene vectors require several characteristics such as high gene condensation; favorable serum stability to avoid non-specific serum protein interactions, endonuclease degradation, and renal clearance; achieved specific targeting cell or tissues; effective transport into the cytoplasm thereby facilitating gene transfection (mRNA, siRNA and miRNA); precise gene release and scheduling, and nuclear localization that enables DNA transcription. Comprehensive exploration of transfection mechanisms can aid in the development of high-performance gene vectors [9, 10].

Gene vectors generally include viral vectors and non-viral vectors. Presently, approximately 70% of clinical gene therapy trials employ viral vectors, which include retroviruses, lentiviruses, adenoviruses, and adeno-associated viruses. Due to their exceptional infectivity, virus-based vectors typically exhibit excellent gene transfection capabilities. However, the clinical safety of viral vectors has been questioned due to their propensity to stimulate immunogenic reactions and induce transgene insertion mutations. Moreover, viral vectors possess several limitations, including low gene loading capacity, inability to deliver large-sized genes, complicated preparation procedures, and the patient cannot be repeatedly administered [4]. In contrast, non-viral vectors, particularly lipid nanoparticles (LNPs) and cationic polymers, have demonstrated robust gene loading capacity, heigh safety and practicability, simplicity preparation [10, 11]. Consequently, non-viral vectors are exhibiting tremendous potential for further clinical development and application. Our review primarily highlights the significant potential of non-viral vectors, particularly lipid nanoparticles (LNPs), highly branched poly(β-amino ester) (HPAE), single-chain cyclic polymer (SCKP), poly(amidoamine) (PAMAM) dendrimers, and polyethyleneimine (PEI). We intend to provide a detailed examination of the latest research progress and existing limitations of non-viral gene vectors over recent years.

## Gene delivery process and the obstacles

DNA and RNA are types of biological macromolecules with large negative charges.

**Fig. 1:**
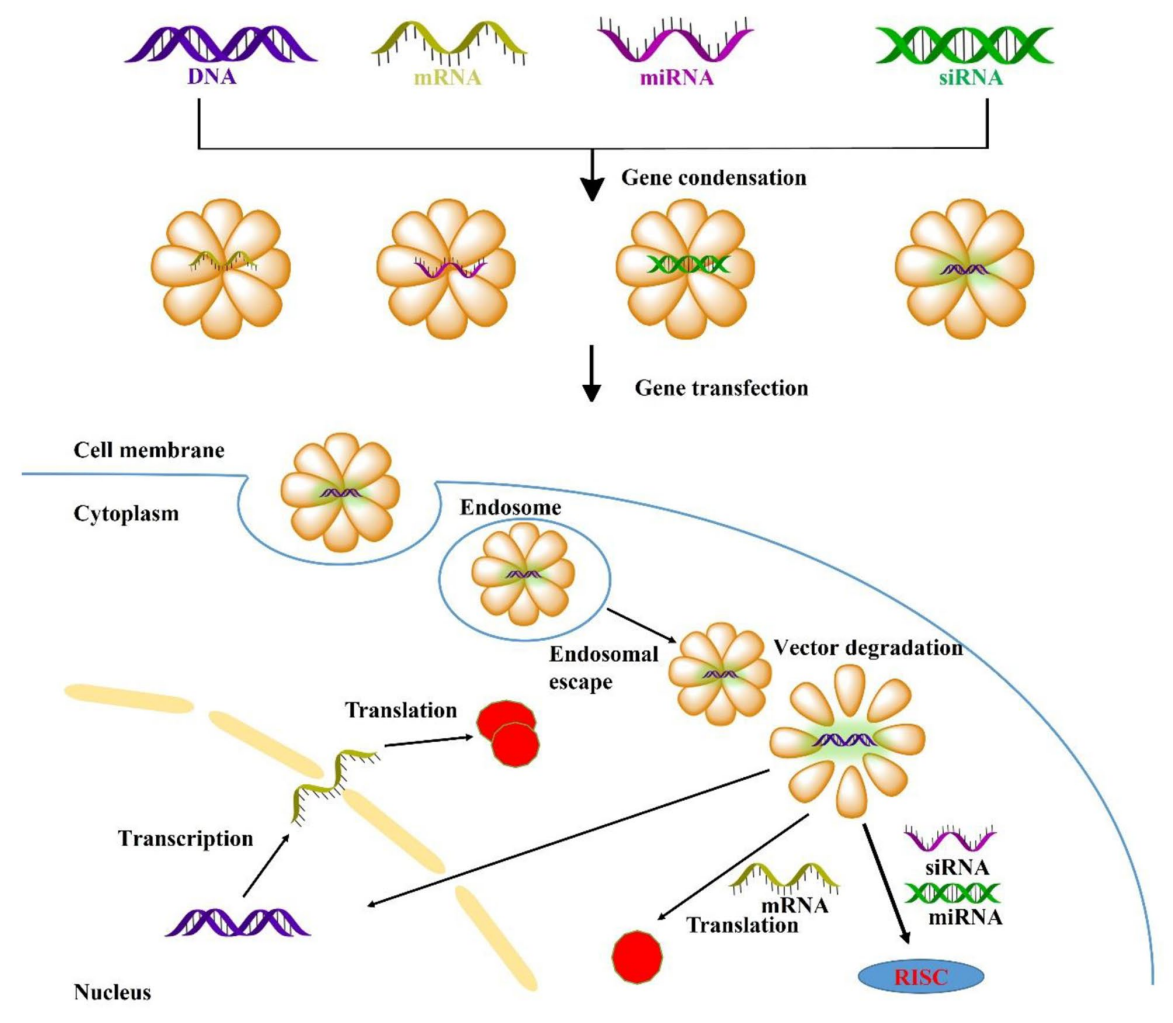
Non-viral vector mediated various gene transfection process, and navigating extracellular and intracellular obstacles for DNA, mRNA, siRNA, miRNA [4]

During gene transfection, there are always multiple extracellular and intracellular barriers during gene transfection that can dramatically influence their gene expression efficiency (Fig. 1), mainly including: (1) DNA and RNA are easily and rapidly degraded by various nucleases in the blood or cells. Furthermore, they are also rapidly eliminated by macrophages and the reticuloendothelial system. The half-life of DNA and RNA in vivo is generally just a few minutes to a couple of minutes [12]. (2) There exist strong electrostatic repulsions between DNA or RNA and the negatively charged phospholipid bilayer, making it quite challenging for cells to uptake the gene [9]. (3) Endosomes have become one of the most formidable intracellular barriers to gene transfection due to their multiple nucleases and low-pH environment [13, 14]. Despite numerous efforts to develop effective vectors for delivering DNA and RNA, their endosomal escape efficiency generally does not exceed 4%. (4) The microfilaments and microtubules in the cytoplasm can significantly affect the transport of DNA and RNA. (5) The nuclear membrane is a phospholipid bilayer that separates the cytoplasm from the nucleus. The nuclear pores are small in size, and small molecules can easily and freely diffuse into the nucleus; however, these pores are highly selective for macromolecules such as DNA and RNA [15]. Exogenous DNA relies on the rupture of the nuclear membrane during cell mitosis to enter the nucleus to complete the transcription. As a result, the utilization of safe and efficient gene delivery vectors remains one of the most promising approaches for effectively overcoming multiple intra- and extracellular barriers during gene delivery.

Effective gene encapsulation represents a prerequisite for successful gene transfection due to the negative charge and size limitations of the gene which can make it difficult for the gene to enter the cell membrane. Cationic vectors can encapsulate gene into nanoparticles ranging in size from tens to hundreds of nanometers through electrostatic interactions or hydrophobic interactions between their large number of cationic groups and the phosphate groups of gene. Compared to linear polymers, the nanoparticles formed by cyclic topology polymers and gene are more compact and smaller size [16]. For example, highly branched polymers, due to their large number of terminal groups, three-dimensional (3D) spatial structure, and strong DNA binding, have exhibited higher gene encapsulation capability compared to linear polymers [17]. Many investigations have demonstrated that nanoparticle sizes that are neither too small nor too large are not favourable for gene transfection. Nanoparticles smaller than 100 nm are more easily endocytosed by cells and easily excreted by cells through cytosolic excretion [13, 18]. In contrast, nanoparticles sizes larger than 300 nm tend to require more difficult cytocytosis for entry into cells and could be removed by cytosolic cells. Additionally, a high positive charge density can lead to significant cytotoxicity and disruption of cellular metabolic activity due to the adsorption of many nanoparticles on the cell membrane surface. Conversely, too low positive charge density is not conducive to cellular uptake of the nanoparticles, leading to lower gene transfection efficiency. Therefore, achieving an optimal positive charge density is crucial for effective gene delivery. Furthermore, if the DNA condensation by the vector is too tight, it is not conducive to the effective release of DNA during transcription, which can result in inefficient transfection.

The topological structure of delivery vehicles, the hydrophobicity of the chain segments, and the size, surface charge distribution, and density of the nanoparticles all have significant effects on their stability [20]. It is necessary to carefully consider these factors to improve gene delivery efficacy and decrease side effects or cytotoxicity. In addition, nanoparticles smaller than 50 nm are usually rapidly cleared by the kidneys, while nanoparticles larger than 300 nm tend to activate the body’s immune response [19]. Therefore, selecting an appropriate size range for nanoparticles is crucial to avoid undesirable side effects [19]. At high physiological salt concentrations, the nanoparticles tend to aggregate, which makes cellular uptake more difficult and even lead to capillary embolism and high toxicity [13]. More importantly, serum albumin and other negatively charged proteins in the bloodstream were adsorbed to the surface of nanoparticles through electrostatic interaction, leading to dissociation or aggregation [13, 15]. These aggregates are easily cleared by macrophages and the reticuloendothelial system, which can impair gene delivery efficiency and reduce the duration of gene expression. Several investigations have indicated that nanoparticles formed by highly hydrophilic polymers with DNA tend to exhibit greater stability under physiological conditions [13–15]. Additionally, nanoparticles with a lower zeta potential exhibited higher stability. This is largely due to the fact that low charge density polymers can form a hydrophilic layer around the nanoparticles, which can effectively block the adsorption of negatively charged proteins on the surface of the nanoparticles. By minimizing protein adsorption and maintaining nanoparticle stability, highly hydrophilic polymer-based nanoparticles may offer a promising strategy for optimizing gene delivery efficiency [13]. Hong et al. demonstrated that a more compact polyethylene glycol (PEG) shell can reduce the interaction of dendritic structured polymer micelles with serum proteins, which significantly improves the serum stability of the polymer (longer half-life) as well as the rate of DNA release [20]. Green et al. also demonstrated that the nanoparticles formed by PEG-modified poly(β-amino esters) (PAEs) condensation DNA can remain stable for long time and exhibit sustained gene transfection for suicide genes [21]. In addition, the density and molecular weight of the hydrophilic chain segments have a significant effect on the stability of the nanoparticles; too high hydrophilicity or too low charge density is not conducive to effective DNA condensation and nanoparticle stability.

Depending on the nanoparticles size, cellular uptake of polyplexes currently follows three pathways: (1) phagocytosis is mainly used to eliminate foreign body particles, which was not an ideal endocytic for gene uptake; (2) microcytocytosis, which mainly involves larger size particle (200 nm-5 μm); and (3) receptor-mediated endocytosis which is currently the most common cell uptake pathway [22]. Receptor-mediated endocytosis is mainly divided into clathrin-mediated endocytosis (CME), clathrin-independent endocytosis (CIE) and macropinocytosis [22]. Liu et al. incubated cells with chlorpromazine (CPZ, CME inhibitor), methyl-β-cyclodextrin (MβCD, CIE inhibitor) and amiloride hydrochloride (AM, macrocytic drinking inhibitor), respectively, and proved that polyethyleneimine (PEI/DNA) polyplexes relied mainly on CME and CIE pathways to endocytosis [23]. Prabha et al. demonstrated that the smaller-sized nanoparticles formed poly(lactic acid-hydroxyacetic acid) (PLGA)/DNA demonstrated higher transfection efficiency than the larger-sized nanoparticles in COS-7 and HEK-293 cell line [24]. Furthermore, owing to the negative charge on the cellular membrane surface, the nanoparticles with positively charged or electrically neutral surfaces are more favorable for cellular uptake. In addition to particle size and potential, the shape of the nanoparticles exhibits significantly impacts the cell uptake efficiency of the nanoparticles. For example, Gupta et al. demonstrated that nanoparticles with rod-like structures exhibit higher cellular uptake efficiency compared to that of disk or spherical morphology [25]. Similarly, nanoparticles possessing a rod-shaped geometry tend to exhibit superior cellular internalization efficiency [26]. Currently, the interaction between surface-specific ligands of the gene vehicles surface, including antibodies, glucose, transferrin, folic acid, transporter proteins and integrin ligands, and specific antibodies/antigens or enzymes/substrates on the targeted cell is the foremost approaches to enhance cellular uptake of nanoparticles [27]. The interaction of ligands on vectors with receptors of cancer cells not only enhances the expression of anti-oncogenes, but also reduces the toxicity on normal cells. As such, targeted delivery and specific uptake of nanoparticles exhibited a broad potential for cancer therapeutics. The guanidine group on the nanoparticle can form multiple hydrogen bonds with polyhydroxy compounds or acetyl heparan sulfate present on the cell membrane, which can promote cellular internalization. Liu and colleagues utilized functionalized modifications to graft guanidinium groups onto the terminal of highly branched poly(β-amino esters) (HPAEs). They demonstrated that these guanidinium modifications significantly enhanced the uptake efficiency of the polyplexes and thus facilitated the gene transfection [28].

On reaching the cytosol, the nanoparticles are wrapped into the lysosomes. Some nanoparticles are expelled from the cell *via* cytosolic spitting, whereas others are transported to the highly acidic lysosomes containing various nucleases [29]. Furthermore, both the naked gene and nanoparticles tended to be rapidly degraded. The lysosome is one of the most challenging intracellular barriers to overcome. For lysosomal escape, two major approaches including “proton sponge effect” and “membrane fusion” have been proposed. Cationic polymers such as PEI, PAE, polydimethylaminoethyl methacrylate (PDMAEMA), and PAMAM mainly rely on the “proton sponge effect” to achieve lysosomes escape [30]. Chloroquine, imidazole, and tertiary amine groups have been grafted onto polymeric vectors to enhance their proton buffering capacity. Liu et al. have designed and synthesized new phospholipid vectors capable of undergoing hydrophobic transitions under different pH conditions. The nanoparticles undergo a transformation from the nuclear shell structure to the hexagonal phase structure, which is prone to endosome membrane rupture, facilitating lysosomal escape [31]. In addition, to effectively inhibit endosome acidification, a pH-responsive PLGA was designed, which could efficiently regulate the escape efficiency of nanoparticles from endosomes in immune cells. Under acidic conditions, the pH-responsive PLGA can release NH_4_HCO_3_ to promote an immune response [32]. After escaping from the endosomes, endosome rupture caused the release of nucleases and other hydrolases from lysosomes into the cytoplasm, which may lead to some cytotoxicity and gene degradation, which reduces the efficiency of gene transfection. The investigators proposed some new pathways for intracellular transport of the nanoparticles, such as endoplasmic reticulum targeting induced nanoparticle to enter the nucleus through the Golgi-endoplasmic reticulum channel, thus avoiding the nanoparticles to enter into the endosome. You et al. have developed an endoplasmic reticulum-targeted gene vector by grafting peptides onto the terminals of cationic LNPs [33, 34]. Their results demonstrated that the endoplasmic reticulum-targeted vector exhibited significantly higher gene transfection efficiency compared to liposome without endoplasmic reticulum-targeting ability.

Once the nanoparticles escape from the endosomes, it will enter into the cytoplasm and be further transported. The cytoplasm contains a variety of proteins, microtubules, microfilaments and other organelles that can impose obstacles to the translocation of the nanoparticles in the cytoplasm. The mechanisms of nanoparticle translocation in the cytoplasm primarily involve two pathways: (1) transport along negatively charged microtubules or microfilaments through non-specific interactions with motor proteins, and (2) redistribution of nanoparticles in the cytoplasm by eukaryotic cells mitosis, thus mitosis plays a major role in the translocation of the nanoparticles in the cytoplasm [4, 35]. Compared to DNA, RNA transfection does not require entry into the nucleus, generates lower immunogenicity, and does not induce mutagenesis. Once siRNA and miRNA released, siRNA is necessary to be loaded into the RNA-induced silencing complex (RISC) to trigger the genetic silence pathway [4, 35]. Therefore, timely release of the encapsulated gene from the cytoplasm is crucial to achieving optimal transfection efficiency. To facilitate gene release, several functionalized modifications have been introduced, such as degradable bonds, pH responsiveness, and modulation of the charge or topology of the vectors [36].

For DNA transcription in the nucleus, DNA released from the nanoparticles must cross the nuclear membrane to enter the nucleus. However, DNA transportation in the nucleus depended mainly on the nuclear pores (9–10 nm in diameter). This size range creates a significant challenge for DNA actively crossing the nuclear membrane into the nucleus [10, 37, 38]. In mitotic cells, cationic polymers tend to exhibit higher transfection efficiency. This is primarily attributed to two factors: (1) exogenous genes rely mainly on cell mitosis to enter the nucleus, and (2) the favorable nuclear localization ability of cationic polymers. However, in clinical trials, gene delivery vehicles need overcome more challenging physiological barriers due to the more sophisticated physiological environment and immune response in vivo.

## Non-viral gene vector

**Fig. 2:**
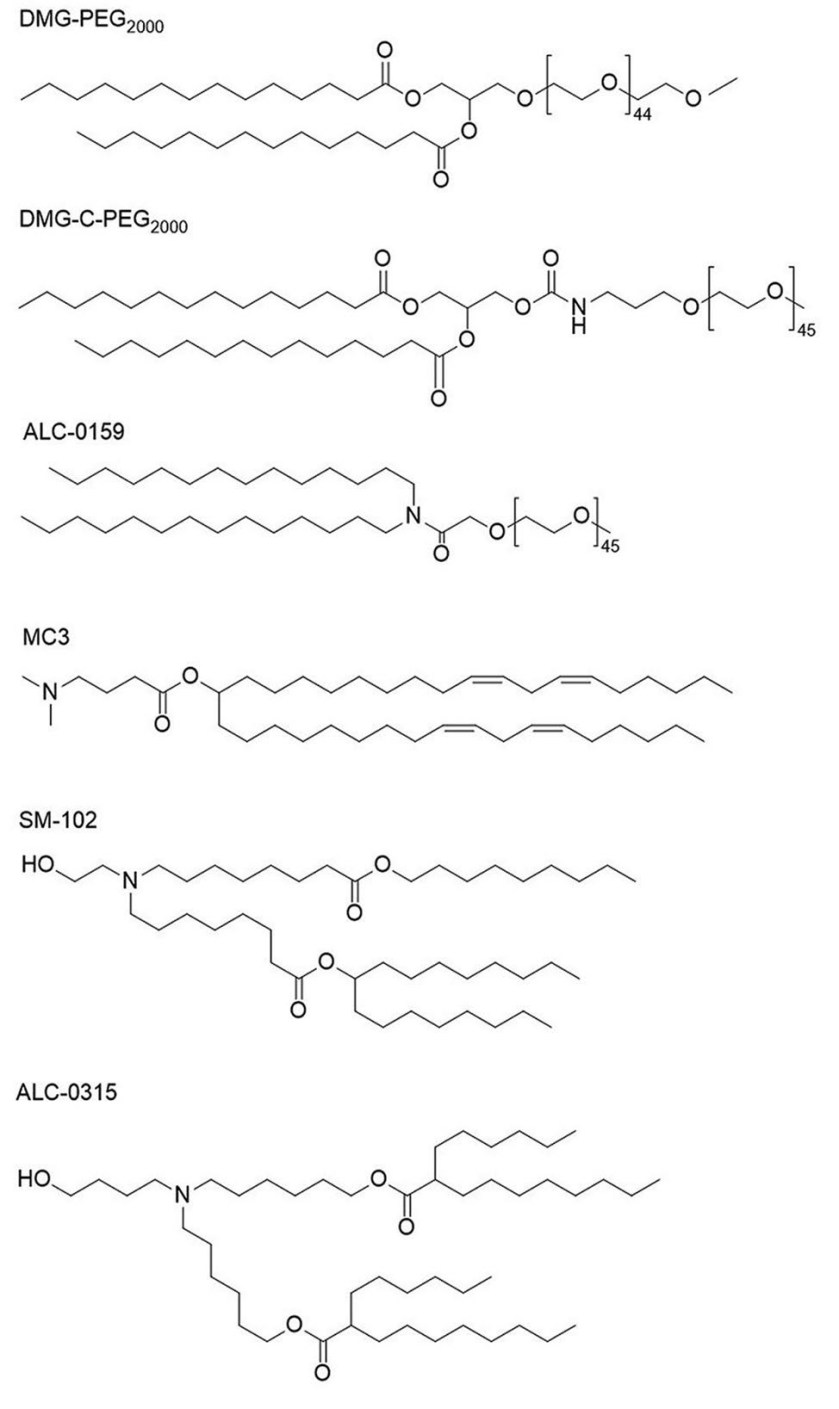
Chemical structures of PEG-lipids and lonizable cationic lipids [35]

**Fig. 3:**
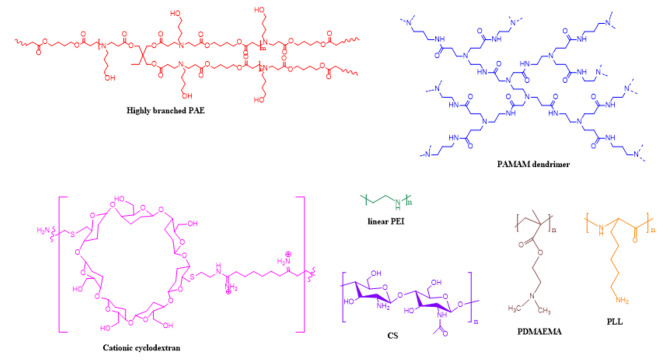
Chemical structures of several common polymer-based gene vehicles [35]

In contrast to viral vectors, non-viral gene vectors offer several advantages including wide raw materials, flexible chemical composition, easily modulated topology, high DNA loading and safety. Non-viral vectors mainly include LNP, exosomes, cationic polymers (such as PEI, PAMAM, PDMAEMA, PAEs, SCKP, polysaccharide macromolecules (chitosan (CS), cyclodextrin (CD) and dextran), inorganic nanoparticles (quantum dots, gold nanoparticles, silica nanoparticles and carbon nanotubes) and polymer hydrogels [39–41]. Currently, LNPs and cationic polymeric based vehicles are the most promising non-viral gene vector candidates. For example, in response to the spread of COVID-19, LNPs-based mRNA vaccines have been urgently approved by the FDA. This chapter is dedicated to the discussion of the most promising non-viral vectors currently under development, which include the following: LNPs, PAE, SCKPs, PEI, PAMAM, PDMAEMA, CS, and CD (Fig. 2 and 3).

### LNPs

**Fig. 4:**
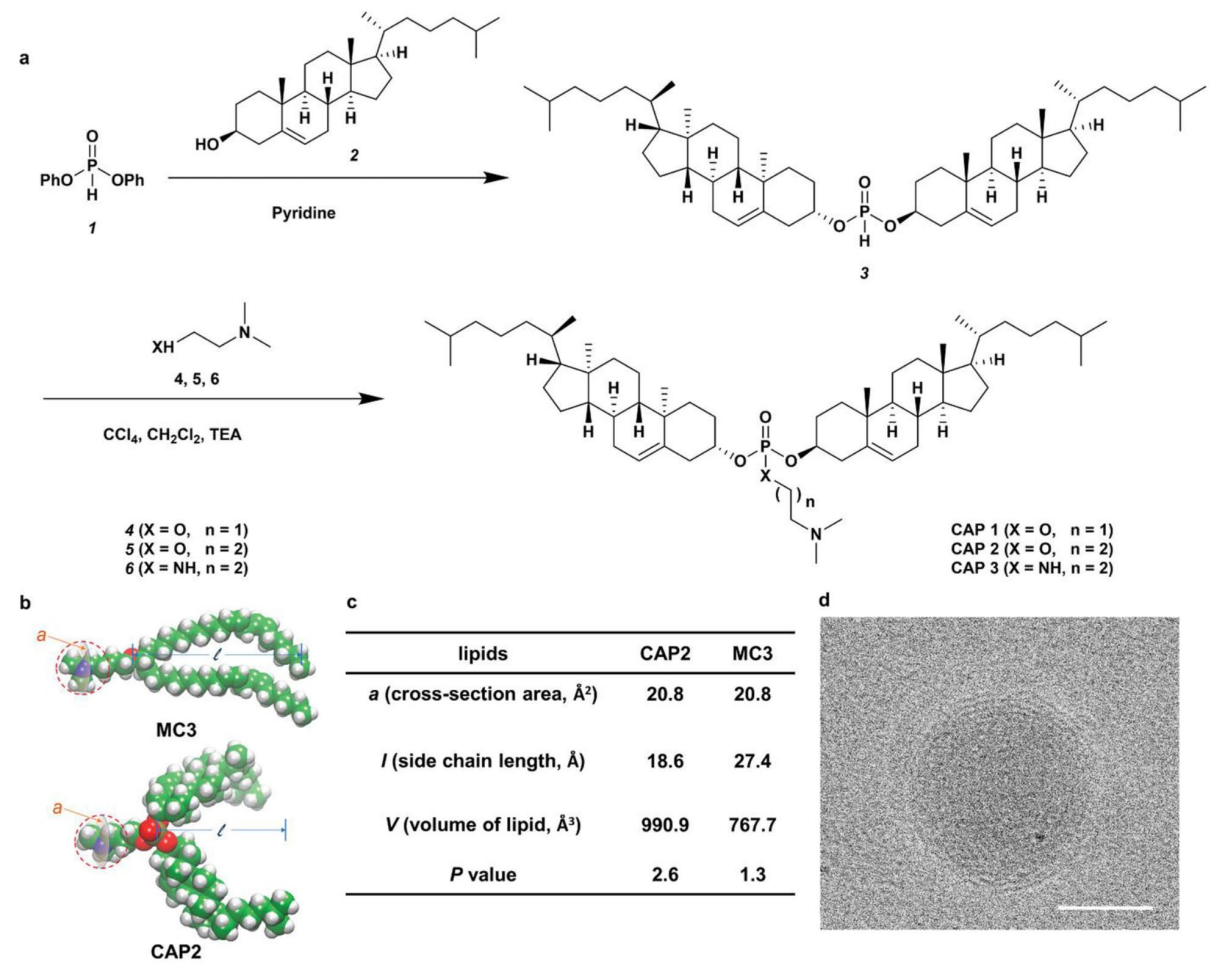
Synthesis of CAP lipids and delivery of mRNA using CAP LNPs [47]. a, synthesis and structures of CAP compounds. b, 3D structures of CAP. C, c, calculated parameters of CAP. d, Representative Cryo-EM image of CAP2-LNPs

LNPs were widely utilized to clinically advance non-viral vehicles for gene therapy. LNPs generally include ionizable lipids (such as N-[1-(2,3-dioleyloxy)propyl]-N,N,N-trimethyl-ammonium chloride(DOTMA), 1,2-dioleoyl-3-trimethylammo-niumpropane (DOTAP), neutral lipids (cholesterol, phospholipid), and polyethylene glycol (PEG) grafted lipid (Fig. 4). Cationic lipids consist of three parts, including cationic head, linking part and hydrophobic tails. Owing to their high load capacity, degradability, practicability, flexible structure and charge, and scalable manufacturing, lipids, particularly cationic lipids, have been widely employed in the delivery of DNA, mRNA, and siRNA. Meanwhile, scientists had exploited many commercial transfection reagents (such as lipofectamine 2000, lipofectamine 3000, DOTMA, DOTAP, and dioctadecylamido-glycylspermine (DOGS) [11, 15] (Fig. 4). Numerous studies have shown that charge density, composition and number of the head and tail, the hydrophilicity or hydrophobicity, the linking group, and the shape (rod or spherical) all plays a major role gene transfection. On18 November 2020, BioNTech-Pfizer and Moderna reported two mRNA vaccines for COVID-19. LNPs are used to deliver mRNA sequences encoding SARS-CoV-2 spike protein to host cells and ultimately to against SARS-CoV-2 infection [42].

According to the current reports, the Medicines and Healthcare products Regulatory Agency (MHRA) has approved two types of mRNA vaccines that utilize LNPs as the delivery vehicles. The mRNA vaccine shows extremely high efficiency (95% and 94.5%). Nonetheless, many severe defects were found requiring harsh low storage temperature, and including few patients with fatigue, headaches, muscle and joint pain, fever and other symptoms after injecting the mRNA-based vaccine [43]. It is likely that due to the poor stability and toxicity of LNPs or body heterogeneity causes adverse reactions. It is possible that adverse reactions of two LNP-based mRNA vaccines may be caused by the poor stability and potential toxicity of the LNPs, as well as the natural heterogeneity of the human body. Compared to DNA transfection, mRNA transfection presents several advantages. Firstly, mRNA does not require entry into the nucleus, and thus does not produce transgene insertion mutations, ensuring higher safety.

Secondly, the transfection process for mRNA is simpler since it involves fewer steps, and there is no need for transcription, leading to fewer barriers for transfection (mRNA does not require crossing the nuclear envelope). Additionally, mRNA enables faster and more efficient kinetics for protein expression. Lastly, proteins are transiently expressed and easily controlled [1, 2, 44].

Currently, LNPs were used as a significant inspiration for designing mRNA vectors, which include long hydrophobic chain ends and positively charged end groups, as well as a linker chain. Recent reports revealed that that the most effective vector has only 4% of the mRNA effective release into cytoplasm [45]. However, endosomal escape has been a significant challenge. Taking inspiration from the natural phospholipids of biological membranes and the structure of cationic lipids, Liu et al. prepared 572 phospholipid molecules that feature pH-switchable, zwitterion, and three hydrophobic tails for mRNA delivery and CRISPR-Cas9 gene editing [7]. Through numerous performance screenings, their results suggest that the length and number of hydrophobic chains, as well as the charges of the phospholipids significant impact on transfection. Optimized phospholipids exhibited a remarkable 965-fold higher in vivo efficiency compared to positive control DSPC. By optimizing these phospholipids, a high-performance composite vector was created that generated high mRNA transfection efficiency and CRISPR-Cas9 gene editing in the liver and kidney, respectively. They emphasized that the length and number of hydrophobic chains effect on the endosomal escape. However, the endosomal escape mechanism of the hydrophobic chain in the transfection process is still not clear. This research is promising research for phospholipid nano-delivery system in the clinical application.

Considering that inhaled mRNA delivery offers great potential in the treatment of lung diseases, Anderson et al. developed a three-component reaction to optimize the linker groups, lipid tails, and heads of ionizable lipid to synthesize the ionizable lipids for inhaled delivery of mRNA and CRISPR-Cas9 gene editing [46]. Results indicated that the ionizable lipids exhibit excellent biodegradability and safety, and can efficiently and repeatedly deliver mRNA for gene editing in lung epithelial cells. Furthermore, to promote phase transformation, endosome escape and the release of the payloads, Dong et al. created cholesterol-amino-phosphate (CAP) lipids RNA delivery systems by incorporating the benefits of cholesterol, phospholipids, and amines [47] (Fig. 4). Ultimately, CAP-LNPs efficiently mediated mRNA encoding DNA meiotic recombinase 1 protein transfection in mouse testis, to rescue the chromosomes recombination and recover the meiosis and spermatogenesis. To increase mRNA vaccine effect, Song et al. constructed hybrid nanoparticles from interferon genes (STING) agonist [manganese (Mn)] and LNPs to promote lysosomal escape and stimulate antigen-presenting cells maturation, and enhance mRNA expression [48]. Despite its promising clinical applications, LNP has some drawbacks, including: (1) the unknown distribution of its four components for LNPs is also a serious limitation; (2) The strong biological inertness of PEG reduces the cell uptake of LNP and mRNA transfection [49]. Compared with the LNPs, the polyplexes, formed by polymers and genetic material, are more stable. The design of the polymer-based vectors may be similar to lipids, including hydrophilic and hydrophobic groups, a balanced distribution of charges, and functional chain groups. Exosomes, like LNPs, are indispensable natural biomaterials involved in the cellular signaling and transfer processes. Exosomes are nano-sized vesicles that contain a variety of biological molecules such as lipids, proteins, miRNA or mRNA, metabolites and other biological molecules. They transfer biological signals between cells to facilitate intercellular communication. Because of their stability in blood circulation, biocompatibility, low immunogenicity, superior coating ability and targeting properties, exosomes are considered a promising and advantageous gene delivery vehicle, particularly for brain tissue and cancer silent therapy. Despite their potential as gene delivery vehicles, there are still many limitations to overcome with exosomes, including the mechanisms and engineering separation.

### HPAEs

In 2000, Langer et al. firstly prepared a small library of linear poly(β-amino ester)s (LPAEs) *via* the A2 + B2 Michael addition reaction of diamine and diacrylate monomers, and showed that LPAEs were one of the most effective non-viral gene vector [50]. As gene therapy continues to rapidly advance, it is becoming increasingly clear that LPAE may not be sufficient to meet the clinical gene therapy. Compared to linear polymers, highly branched polymers have three-dimensional structure and multiple functionalized sites, previous investigations have clearly illustrated branched polymers such as PEI [51], PAMAM [51], PDMAEMA [52] all emerged higher transfection efficiency compared to linear analogue. Thus, HPAEs have several obvious advantages for exploiting the high-performance gene vectors. In the past, HPAEs were typically prepared *via* the “A2 + BB’B” Michael addition method, which often required special monomers or harsh reaction conditions such as special catalysts or high pressure [53]. These methods were not ideal for advancing non-viral gene therapy towards clinical application.

In 2015, Zhou et al. firstly exploited one-pot “A2 + B3 + C2” Michael addition strategy to design and synthesize a small library of HPAEs (Fig. 3), and proved that HPAEs, as one of the most effective non-viral vectors, show superior transfection efficiency and great promise in the clinical treatment [54]. Through optimization of their chemical composition, such as amine monomers, diacrylate monomers, and end-capping monomers, 22 HPAEs with superior performance were prepared. The researchers systematically investigated the relative DNA binding ability, size and zeta potential, morphologies, transfection efficiency and cytotoxicity of HPAEs/DNA polyplexes. The results demonstrated that HPAEs exhibited higher transfection efficiency than commercial transfection reagents such as PEI, SuperFect, and Lipofectamine 2000 in 12 different types cells such as HKC8, COS7, 3T3, NHK, RDEBK, HeLa, HepG2, rADSC, hADSC, SHSY-5Y, Neu7, and primary astrocytes, especially the Gluc activity of the optimized HPAE is higher up to 8521-fold higher than the LPAE analogue. Recessive dystrophic epidermolysis bullosa (RDEB) is a devastating genetic disease caused by a lack of anchoring dermis and epidermal collagen VII (C7). HPAE can effectively mediate COL7A1 gene encoding C7 into keratinocytes and fibroblasts, in RDEB graft mouse. More importantly, immunofluorescence image clearly observe that the C7 collagen is localized along the base membrane zoon and persisted to restore C7 expression and secretion for 30 days, demonstrated that HPAE have great prospect for clinical treatment of RDEB [55].

**Fig. 5:**
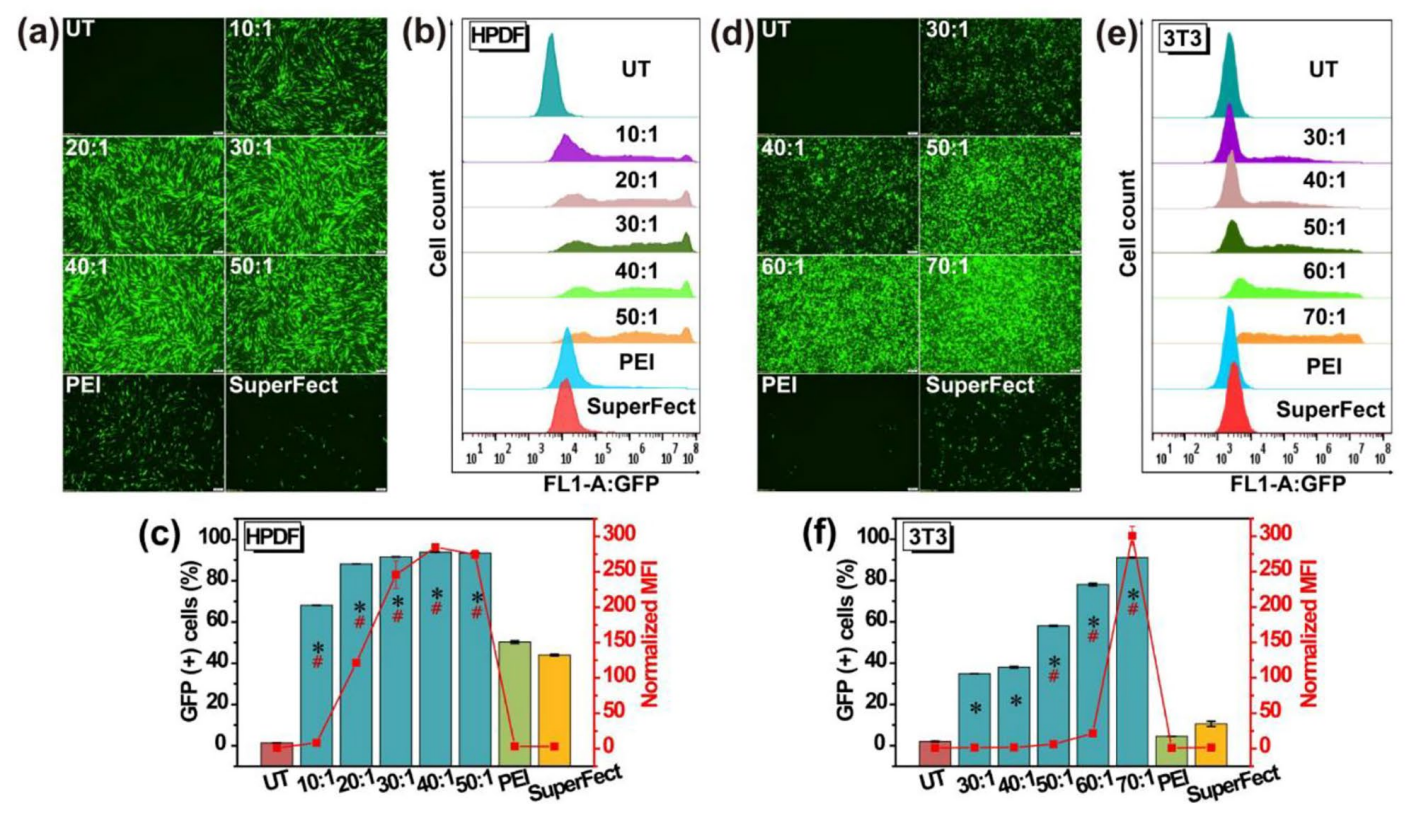
Representative LBPAEs show great superiority in gene transfection in different cell types [56]

To explore the properties relationship between linear and highly branched structures of PAEs, Zeng et al. developed a new type of linear-branched poly(β-amino ester) (LBPAE) for gene delivery by linking oligomeric LPAEs with branched monomers [56] (Fig. 5). The researchers evaluated the biophysical properties of LBPAE, including polyplexes size, zeta potential, DNA condensation, cellular uptake, proton buffer capacity, DNA release, and degradation. Results demonstrated that LBPAE exhibited ultra-high Gluc and GFP expression in human primary dermal fibroblasts and mouse embryo fibroblasts. The study clearly revealed highly branched structure can significantly enhance the transfection efficiency of PAEs. Using similar methods, researchers have also introduced branched monomers into other linear macromolecules such as PEI, PAMAM, CS, and PLL to construct other branched macromolecule-based gene delivery vectors. This suggests that the development of branched macromolecule-based gene delivery vectors may be a promising avenue for achieving higher gene transfection efficiency in future gene therapy applications.

In addition to the modifications of introducing branched monomers, there have also been extensive studies on numerous functional modifications of HPAEs. These include modifications for external responsiveness, targeting, degradability, and hydrophilic and hydrophobic balance. For instance, to facilitate gene delivery in difficult-to-transfect cells, Liu et al. developed thermos- and pH-responsive HPAEs by altering the chain length of PEG and the number of amino groups, which allows for responsive release of the therapeutic genetic material [57]. More importantly, many functional modifications of HPAEs including external responsiveness, targeting, degradability, hydrophilic and hydrophobic balance were also studied. For difficult-to-transfect cells, Liu et al. prepared thermos- and pH- responsive HPAEs by altering the chain length of PEG and the number of amino, to achieve responsive release. Subsequently, HPAE with disulfide backbone are synthesized to facilitate the degradation and, and the glutathione were used to achieve rapidly and targeted releasing DNA for cancer cells and reducing the cytotoxicity [58]. In addition to the modifications mentioned earlier, HPAEs grafted with guanidine groups have also been developed recently. These guanidine groups can form multiple hydrogen bonds with the cell membrane, which enhances cellular uptake and ultimately leads to efficient cancer gene therapy [28]. Another promising modification of HPAEs is the functionalization with folate. Folate is a vitamin that is taken up by many cancer cells through the folate receptor, which is often overexpressed in many types of cancer cells. HPAEs functionalized with folate have exhibited robust gene transfection efficiency and cancer cell targeting ability, resulting in improved gene therapy efficacy. These responsive HPAEs offer the potential for improved gene transfer efficiency and site-specific gene delivery in challenging cell types in the future.

Indeed, the functionalization of HPAEs with a single modification may limit their potential and applications in gene therapy. Multi-functional HPAEs can be designed and synthesized by incorporating multiple functional groups into the same polymer chain. For instance, Liu et al. have designed and prepared multi-functional HPAEs with superior transfection efficiency and low cytotoxicity for difficult-to-transfect cells [17]. They used an iterative optimization approach that involved three aspects: branched structure, degradation, and cell uptake. This allowed them to screen and identify HPAEs with optimal properties and functionalities. The screened HPAEs exhibited superior transfection profile efficiency for difficult-to-transfect cells (77% and 52% for ADSCs and astrocytes, respectively) and low cytotoxicity, and was found to mediate high-level expression of nerve growth factor in astrocytes, and induce the growth of nerve protrusion. In addition, the investigation illustrated that the iterative optimization of multiple functional monomers appears to be a versatile method for the development of optimized multi-functional polymer-based vectors. However, it is important to note that the incorporation of multiple functional monomers can lead to steric hindrance and performance differences between the different functional groups, which may result in unfavorable restrictions for the design and development of these polymers. The synthesis of multi-functional polymers with multiple functional groups provides new opportunities for the design and engineering of advanced gene delivery systems. However, it is still challenging to control the molecular architecture and properties of these polymers due to the complexity of incorporating multiple functional groups. As one of the most important branching parameters in the synthesis of HPAEs, and it is crucial to understand how different branching strategies can impact transfection performance. We employed two different branching strategies (“A2 + B3 + C2” and “A2 + B4 + C2”) to synthesize a series of HPAEs and evaluated their transfection efficacy [59]. Our study assessed polyplex size and zeta potential, DNA condensation, cell uptake, transfection efficiency, and cytotoxicity. we revealed that the HPAEs synthesized via the “A2 + B4 + C2” strategy had a higher degree of branching, resulting in improved DNA condensation and cell uptake efficiency, which found that the branching strategy had a significant impact on DNA transfection performance. The transition of HPAEs from the laboratory to the clinic is a critical step in realizing their therapeutic potential. Clinical trials will be necessary to assess the safety and efficacy of these gene delivery systems in humans. Additionally, while most studies have focused on the delivery of DNA, there is a need to develop HPAEs for the delivery of other types of genetic material such as mRNA, miRNA, or proteins. Firstly, attributed to the different transfection process and mechanism to various biological macromolecules, the new types HPAE are redesigned from structure and composition to functional monomer, to meet the personalized delivery requirements. Secondly, the designing novel vectors can simultaneously deliver multiple genetic material or gene/drug composited system, as well as integrated diagnosis and treatment system.

Based on non-invasive aerosol delivery genes in the lung diseases treatment, Patel et al. used A2 + B2 + BB′2 to prepare HPAEs via Michael addition for luciferase mRNA delivery [60]. The optimized HPAEs displayed robust transfection efficiency, especially the high luciferase expression in the lungs via nasal nebulization. The study proved that HPAEs efficiently mediated inhaled mRNA delivery, and provided a considerable platform to mRNA and miRNA delivery. To reduce the in vivo toxicity of PAEs and further investigate their clinical therapeutic potential, Philip et al. synthesized a novel a branched poly-β-amino-thio-ester, demonstrated that HPAE had excellent safety profiles in a variety of animals and efficiently mediated delivering the Cas13 mRNA and crRNA transfection in the hamster model. Finally, SARS-CoV-2 RNA knockdown in the lungs was significantly reduced by 81.9%. These findings demonstrate that the HPAEs vectors exhibited great clinical applicability for gene therapy [61]. Based on the uncertainty charge of cytoplasmic proteins, Rui et al. designed and synthesized a HPAEs with positive and negative charges to delivery different type proteins including saporin, BSA, GFP [62]. For intracellular proteins, many biophysical characteristics, including DNA condensation, lysosomal escape and transfection were thoroughly evaluated. Moreover, the optimized HPAE can achieves high level CRISPR gene editing via delivering Cas9 RNP. More importantly, its GFP knockout rate achieved 77% in HEK cells and local gene knock in the mouse brain [62]. Therefore, the amphoteric charge of HPAE provides a versatile and effective platform for the cytoplasmic protein delivery, especially CRISPR-Cas9 gene editing in the brain diseases and cancer treatment.

Although numerous studies have demonstrated the extensive application prospects of HPAE in gene therapy, there exist several facets that warrant further examination, such as: (1) The precise preparation of various topological structure of PAE, namely dendrimers, stars, brushes, and cyclic structures, and the corresponding analysis of the influence of these topological structures on transfection efficacy. (2) Developing a multifunctional PAEs, such as responsive degradation release, visual detection, and multi-gene co-delivery platform. For charge-altering releasable polymer-based vectors, Mckinlay et al. introduced an innovative method based on rapid and selective degradation of poly(α-amino ester)s for effective mRNA delivery through pH-induced degradation. This approach enables quick discharge of mRNA and DNA in the cytoplasm by eliminated the charge of the transportation vehicle [36]. The study’s methodology of manipulating charge and triggering mRNA release through degradation is ingenious and innovative. However, the complexity of preparing special monomers hinders the feasibility of large-scale production. (3) Currently, Most of HPAE research focuses on in vivo and in vitro, so extensive clinical trials are essential to advance the next generation of research.

### Cyclic polymer and single chain cyclic polymer

Owing to the distinctive topology of the cyclic polymer such as tadpole-shaped, sun-shaped. it has exhibited many unique physicochemical properties, such as structural and size tunability, notable coating ability, low cytotoxicity and controlled release capabilities. Taking into account the impact of topology on its gene transfection performance, Cheng et al. prepared the sun-shaped PDMAEMA to deliver DNA [63]. Preliminary cell experiments confirmed that the sun-shaped PDMAEMA has high transfection efficiency. Although lacking verification through animal testing, this research offers evidence that the sun-shaped or cyclic polymer vector has the potential to emerge as the next-generation high-performance gene vector. Currently, the difficulties of synthesis and purification of larger-scale batches still limited the application and development of cyclic polymers, so there are few reports about cyclic polymers in gene delivery. Wei et al. employed ATRP and click chemistry to synthesize cyclic PDMAEMA, which was firstly applied to gene delivery [64]. Their findings initially revealed that the cyclic PDMAEMA exhibited comparable transfection efficiency and lower cytotoxicity in HeLa cells in comparison to its linear counterpart. This pioneering study provides insight into the potential of cyclic polymers in gene delivery applications. Similarly, cyclic PEI showed higher transfection performance and lower cytotoxicity compared to linear PEI under the same molecular weight [65]. Compared with branched PEI, cyclic PEI exhibited a more compact structure, which results in superior overall transfection performance and lower cytotoxicity in HFF-1 cells and HAE cells. Therefore, we conjecture that cyclic poly(β-amino esters), PAMAM, PDMAEMA and PLL may be exploited for effective DNA or mRNA delivery. For the tighter structure of the cyclic polymer, they may also be employed for transporting the small molecules nucleic acid including siRNA and miRNA. Since the synthesis of high-purity cyclic polymers always faces great challenges, there is a critical need for conducting more in-depth and extensive research on these polymers. Based on the understanding of cyclic structures, sunflower-shaped, tadpole-shaped polymer, as well as inspiration from the most common genetic material vector sperm cells, we speculate that tadpole-shaped polymers may possess superior transfection capabilities. It is possible that sperm cells’ unique topological structure enables deliver genetic material into egg cells, and designing a sperm-like (tadpole-shaped) vehicle may yield unexpected advantages for gene delivery.

In recent years, single-chain cyclic structure, as a new beginning type of nonlinear structure, is proposed. During their extensive research on multivinyl monomer polymerization, Wang et al. found that the critical gel point of branched polymers is always higher than the predicted point according to F-S theory [66]. By employing degradable multivinyl polymers, they found that the SCKP’s d the branching degree and molecular weight did not significantly decrease after degradation [67]. On the contrary, the F-S theory predicted that the polymer chain will be broken, leading to the molecular weight significantly decreasing after degradation.

**Fig. 6:**
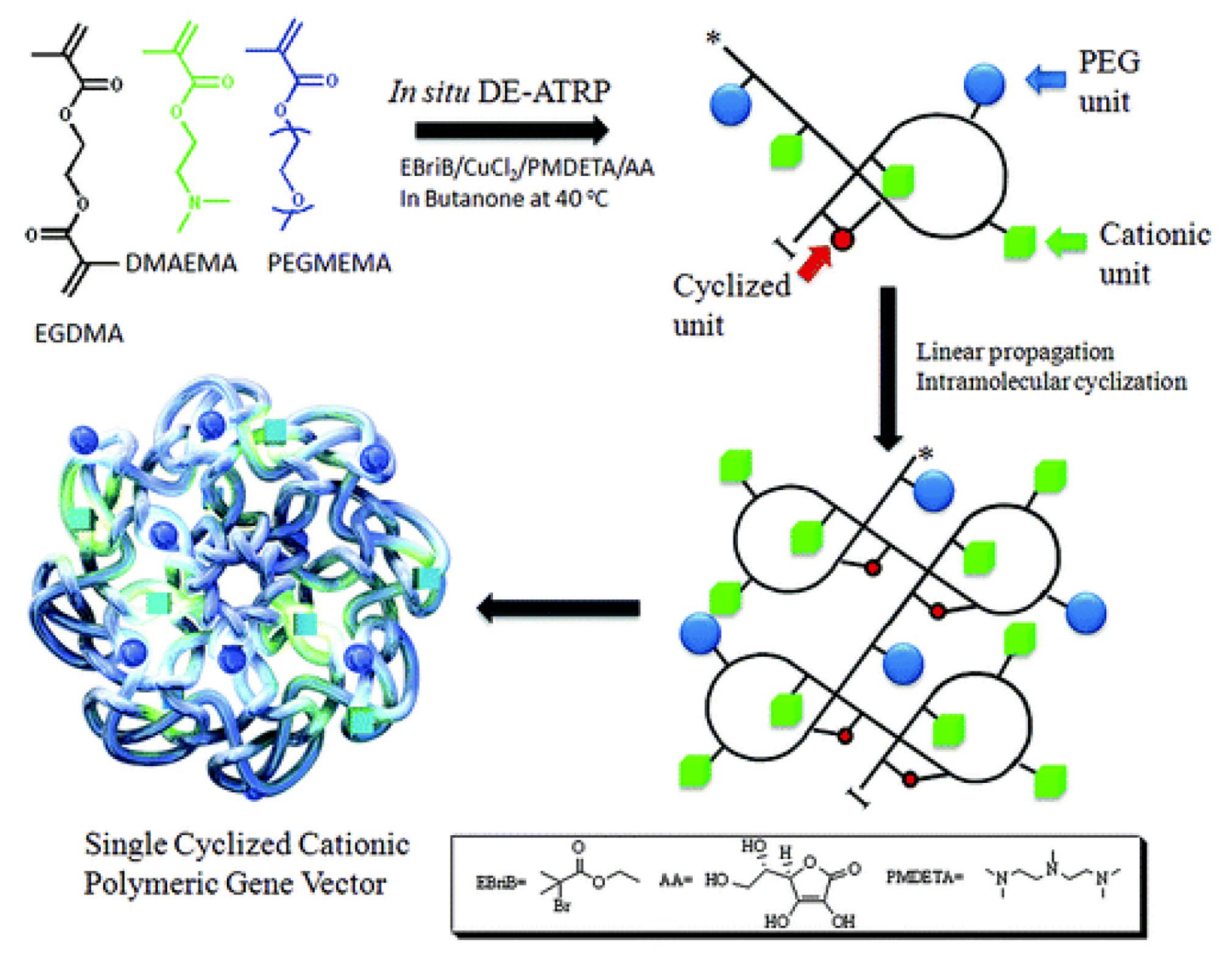
Synthesis of SCKP via in situ DE-ATRP reaction and graphical illustration of cyclized knot structure [68]

It is hypothesized that multiple internal cyclization events occurred inside the polymer, which effectively inhibited intermolecular cross-linking and delayed the critical gel point, during the polymerization of the multivinyl monomer. Through various experimental support and data analysis, they clearly verified single-chain cyclic structure, thereby supplementing and revised the F-S dynamic theory. By utilizing a kinetic control strategy to regulate intramolecular/intermolecular cross-linking reactions under high concentration conditions, Wang et al. successfully synthesized a novel SCKP *via* in situ deactivation-enhanced atom-transfer radical polymerization (DE-ATRP). In 2012, leveraging the unique characteristics of SCKP, Newland et al. utilized deactivation enhanced ATRP to effectively impede crosslinking between multivinyl monomers and promoted the single chains cyclized reaction to synthesize SCKPs for DNA delivery (Fig. 6) [68].

This research preliminarily confirmed the novel SCKP vector exhibits superior transfection profile and cell viability, and can replace the current low-efficiency and high-cytotoxic dendrimer-based vector. Due to the high cytotoxicity of the SCKP resulting from the poor degradation ability, Newland et al. utilized disulphide intramolecular crosslinks to achieve SCKP [69]. Their subsequent work demonstrated that the degradable SCKP based-nanoparticles displayed high transfection in astrocytes, compared to PEI, SuperFect, Lipofectamine 2000, and preserve 80% cell viability. In order to further improve the degradation performance, Gao et al. utilized ring-opening addition polymerization and RAFT to synthesize a main chain degradable SCKP for DNA delivery. Their findings showed that the backbone-degradable SCKP achieved high transfection efficiency and low cytotoxicity in 3T3 and HeLa cells, compared to non-degradable polymers [70].

Compared to dendrimer and topological structure polymers, SCKPs have demonstrated several advantages in gene transfection due to the simple chain synthesis technology and the wide availability of vinyl monomers. Furthermore, real-time and fast gene release may be adjusted by altering the knotting’s ratio, which can greatly improve gene transfection capacity. Although SCKPs excel in transporting DNA at the cellular level, their transfection performance in vivo remains unexplored.

Moreover, SCKP nanoparticles may also be useful for delivering other types of genetic material such as siRNA, mRNA, and miRNA. SCKPs have enormous potential in precision nanoparticles and personalized treatment. The size, batch, and quality of the gene and vector nanoparticles can be effectively controlled, which can achieve precise nanoparticles delivery and precise therapy for individuals and overcome differences dosing between heterogeneous bodies.

### PEI

since 1995, PEI (Fig. 4) has been considered the one of most promising gene delivery vectors [71]. Because PEI contains one nitrogen atom in every three atoms, and branched PEI have numerous primary, secondary and tertiary amines at its unique structure [15, 72]. PEI generally exhibit high gene condensation through strong electric charge, proton buffering ability to trigger endosomal escape, and superior transfection characteristics. In fact, linear PEI (25 kDa) is often considered the gold standard for transfection and serves as a common positive control in vitro transfection. Currently, despite high molecular weight PEI exhibits high transfection efficiency, powerful positive charge and non-degradable structures of PEI lead to high cytotoxicity, while low molecular weight PEI is low cytotoxicity, but its transfection efficiency is always too poor, which severely hinders PEI application. Despite these challenges, PEI-based gene vectors still cannot meet the clinical trials. To address this challenge, various methods have been explored to strike a balance between transfection efficiency and cytotoxicity, such as: (1) introducing degradable groups (such as disulfide bonds) into the PEI backbone to improve its degradation; (2) low molecular weight PEI are cross-linked to synthesized to enhance transfection property; (3) functionalized monomers are covalently grafted to the terminal amino groups to improve targeting and release. 4. PEI composited with other type of materials to develop high-performance composited vectors. Chen et al. constructed a high-performance PEI-graphene oxide (GO) composited gene vector via carboxyl reacting with amino. Finally, branch PEI-GO exhibited lower toxicity and higher transfection efficiency compared to PEI (25 kDa) [73]. Similarly, many PEI-based composited vehicles such as PEI/carbon nanotubes, BPEI/quantum dots, BPEI/nanohydroxyapatite particles, PEI/nanosilicon particles, can also be constructed for various applications. Single two-phase compound is difficult to meet various application requirements, so the multi-component PEI composited system may be considered as a promising research direction. Hu et al. synthesized conjugation of indolicidin to PEI, and demonstrating that hydrophobic chain can enhance transfection efficiency of PEI [74]. In addition, Zhang et al. linked adamantane-BPEI and polyaspartamide with disulfide bonds via the host and guest interaction of cyclodextrin and adamantane to construct a biodegradable nano-delivery supramolecular vector [75]. The new degradable nano-supramolecular delivery system combined with the advantages of multiple components, reduced cytotoxicity and promoted cellular internalization and gene transfection, which provides a major reference platform to constructing the nanoparticle-based gene vehicles. Cook et al. synthesized a new hyperbranched poly(ethylenimine-co-oxazoline) via the thiol-yne reaction and acid hydrolysis [76]. They demonstrated that hyperbranched poly(ethylenimine-co-oxazoline) retained lower cytotoxicity, yet its transfection efficiency was slightly lower than gold standard. Thus, block hyperbranched PEI still needs further modification to improve its transfection efficiency and meet diverse application requirements. Inspired by the topological structure advantages, Huang et al. reported a novel star polymer via arm first method for gene delivery vectors [77]. LPAE was first prepared via Michael addition from 5-amino-1-pentanol and 1,4-butanediol diacrylate and then reacted with branched PEI to form the star polymer. This star polymer exhibited low cytotoxicity and high gene transfection efficacy, demonstrating the successful combination of the advantages of various structure polymers (low toxicity and high transfection) to design a star-shaped polymer vector. Various studies have highlighted that branched polymers have many significant advantages in gene therapy, but there are still many shortcomings, such as poor structural precision and few systematic studies, which hinder PEI-based vectors in clinical practice. Perhaps it is very meaningful to study on branched polymer vectors, including (1) precisely preparing various functionalized branched PEI; (2) constructing an organic-inorganic PEI-based nanoparticles; (3) developing in-depth animal experiments and pre-clinical experiments.

### PAMAM dendrimer

PAMAM dendrimers (Fig. 4) are perfectly symmetrical spherical, actinomorphous polymer. PAMAM dendrimers, due to their large number of secondary and tertiary amines, exhibit strong condensation and proton buffering capabilities, leading to superior gene transfection performance [9]. Numerous reports have proved that the generation number of dendrimer determines its transfection efficiency, and partially degraded dendrimer always shows higher transfection efficiency [8]. PAMAM dendrimers, due to their defined dendritic structure and extremely low polydispersity index (PDI), hold great promise for precision treatment. However, several challenges including complicated preparation, inability to mass production, poor degradation, and high biological toxicity, all hinder the clinical translation of PAMAM dendrimer. The commonly modified methods for dendrimer included a terminal functionalization, the construction different topologies and designing PAMAM dendrimer/nanoparticle composited system. Liu et al. designed a boronic acid-grafted PAMAM dendrimer for protein delivery based on nitrogen-borate complexation and cation-π and ionic interactions between the polymer and protein [78]. Cell experiments demonstrated that the boronic acid-grafted dendrimer vector had good delivery ability for multiple proteins, including fluorescent proteins, β-galactosidase, saporin, ribonuclease A and trypsin, and Cas9 ribonucleoprotein. The boronic acid-grafted vector can basically overcome delivery restrictions caused by uncertain charge proteins, which developed a versatile platform for CRISPR-Cas9 gene editing or other types of proteins delivery. Currently, non-linear topological polymers, especially hyperbranched polymers, are mainly focused on the various genetic materials/small molecule drugs delivery, there is few reports about hyperbranched polymers delivery proteins. However, it is possible that boronic acid-grafted hyperbranched polymers could be developed for protein delivery. From the similar designing inspiration, Wang et al. utilized fluorine grafted PAMAM dendrimer terminal group to deliver DNA and siRNA [79]. Considering that multiple functional modifications caused the serious steric hindrance, Hong et al. developed a facile strategy to graft guanidinobenzoic acid containing guanidyl and phenyl bifunctional group on PAMAM dendrimer to deliver genes DNA and siRNA [80]. Meanwhile, the guanidyl group of the PAMAM dendrimer facilitates nucleic acid condensation, while the phenyl group enhances endosomal escape [81]. Therefore, dendrimer terminal grafting functional group or molecule is crucial for expanding its application. There are still some problems, such as many single functional monomers leaded to the space obstacle. Perhaps the terminal group grafting with oligomers or polymer can solve this problem, but it will inevitably cause the shielding some function. However, the non-degradability of PAMAM dendrimers still faces obstacles. degradable PAMAM dendrimers were designed through the optimized main chain components, such as introducing a certain proportion of disulfide bonds.

Although the miRNA’s mechanism is not yet fully understood on tumor dormancy, there have been related preliminary research for tumor silencing treatments. For example, Tiram et al. employed dendritic polyglycerolamine to deliver miRNA for targeted treating osteosarcoma [82]. The polyglycerolamine-loaded miRNA effectively inhibited the growth of osteosarcoma blood vessels in vivo and in vitro. Furthermore, the polyglycerolamine-loaded miRNA forms dormant state with osteosarcoma tumors to achieve tumor suppression. Their investigation comprehensively explained the mechanism of miRNA in tumor treatment from in vitro to in vivo, and offered a versatile platform for miRNA in the clinical treatment of osteosarcoma using miRNA. Likewise, Song et al. exploited phenylboronic acid gifted on PEG-PAMAM dendrimer to deliver miRNA for the gastric cancer treatment. In vitro experiments indicated that PEG-PAMAM dendrimer/miR-34a could inhibit the proliferation, migration and invasion of cancer cells, and exhibit superior anti-tumor effects [83]. Taking advantage of the structural benefits of dendrimers and star-shaped polymers, PAMAM dendrimers have been exploited as a core to prepare star polymers via grafting to linear polymers. However, the limited number of arms on star polymer vectors results in low cargo capacity, which limits the application of star polymers. There are few comprehensive and systematic researches including many factors, such as molecular weight, chemical composition, hydrophilicity and hydrophobicity, charge density, and specific targeting. It is arduous to truly develop the star-shaped polymer vector and achieve optimal performance.

Further research about star polymer vectors may mainly include: (1) Developing high-performance polymer involving with high active sites, visualization, and biodegradable nuclear; (2) constructing various structures, including multifunctional arms and precise hybrid arms; (3) exploring the collaborative research of core and arm structures for enhanced performance; (4) exploiting complex structure systems of star polymers and other topological structure polymers. Since star polymers with one core may not overcome all hurdles in gene therapy, developing multi-core structure polymers may offer a new avenue for research. Hyperbranched polymers have a similar structure to the precise linkage of many star polymers, providing many structural advantages in gene delivery.

### Other common non-viral gene vectors

Cyclodextrin (CD) (Fig. 4) is naturally-occurring cyclic oligosaccharide molecule. Owing to variations in the number of linked glucose units, CDs are categorized into α-, β-, and γ-CD, respectively. As the most representative cyclic oligosaccharide, CD possesses good biocompatibility, cargo performance and multiple reactive sites. The modified CDs have been widely utilized to gene delivery [84, 85]. Therefore, the first clinical trials of non-viral gene therapy were conducted with CD-based vectors. However, duo to the lack charge, the DNA condensation and proton buffer capacity of CD is always poor, resulting in low transfection efficiency [86]. Several methods are currently being developed to improve the transfection efficiency of cyclodextrin vectors, including: (1) grafting cationic oligomers onto the surface of cyclodextrin to enhance positive charge; (2) utilizing cyclodextrin as a core to construct a star-shaped vector that enhances transfection efficiency through topological structure; and (3) Modulating the terminal groups of CDs, such as cancer cell targeting or supramolecular self-assembly. Chen et al. constructed pH-responsive cyclodextrin integrated PEI hybrid nano-vector for antisense oligonucleotide delivery [87]. Their research indicated that the CD-based hybrid vector exhibited higher transfection efficiency and lower cytotoxicity than PLGA-based counterpart, branched PEI (25 kDa) and Lipofectamine 2000. Additionally, it was found to efficiently mediate antisense oligonucleotide to inhibit tumor cell growth and promote cell apoptosis. Based on the advantages of star topology polymer, Loh et al. utilized CD as core and PDMAEMA copolymer as arm to develop star CD-based vector [88]. Their research revealed that topological structure plays a major role in enhancing transfection performance. Considering the CD topological structure, it may further prove that cyclic polymers (monocyclic or polycyclic polymers) also have broad gene delivery prospects.

Chitosan (CS) (Fig. 4) is a positively charged, biodegradable polysaccharide typically obtained through the deacetylation of chitin. CS boasts several benefits, including low toxicity, low immunogenicity, excellent biocompatibility, a range of modified sites, making it a popular choice for gene delivery efforts [89]. The molecular weight and the degree of deacetylation of CS are the major parameters for the transfection property. In addition, there are still many limitations such as easy aggregation, weak proton buffering capacity, and low transfection efficiency. Tripathi et al. leveraged the benefits of both PEI and CS to create a targeted gene vector through their conjugation. Their research demonstrated significant gene expression in mouse peritoneal macrophages [90]. Inspired by similar inspirations, Zhao et al. designed a novel biodegradable CS-based vector by disulfide bonds to link CS and PEI [91]. The novel CS-based vector mediates the BMP2 gene to effectively promote alkaline phosphatase activity and mineralization in osteoblast cells and stem cells. Universally acknowledged, the functionalization is still relatively single, so multi-functional CD-based vectors should be comprehensively developed from many perspectives, including charge density, targeting, and topological structure.

Due to its superior water soluble, DNA condensation and proton buffering capabilities, PDEMAMA (Fig. 4) has been utilized as one of the earliest cationic polymer-based gene vehicles [92, 93]. However, PDMAEMA is prepared via free-radical polymerization of vinyl monomers leading to its poor degradation and high cytotoxicity, which severely limits in clinical application [94].

PLL (Fig. 4), homopolypeptide, has been widely reported as a gene vector. Unlike other cationic polymers, PLL lacks tertiary amines, which results poor proton buffering capacity and weak endosomal escape [95]. Usually, the chloroquine or cationic groups modified PLL can improve endosomal escape efficiency [96]. In addition, other modification methods involving folate targeting, antibodies, and other strategies have been utilized to graft PLL. Nevertheless, numerous reports have highlighted that PLL cannot achieve high-efficiency transfection and diversified clinical application, and general be used as a positive control in cell experiments [9].

## Translational and clinical research of non-viral gene therapy

Considering the advantages of gene therapy in the treatment of radical and incurable diseases, as of 2023, several gene therapy products have been approved or clinical trials of gene therapy, mainly including the treatment of genetic disorder such as spinal muscular dystrophy, hemophilia B, beta-thalassemia, hereditary hearing loss, RDEB [41, 97, 98], vaccines against infectious diseases such as COVID-19, malaria, Ebola [5, 44], cancer treatment such as bladder cancer, melanoma, ovarian cancer, and pancreatic cancer [1, 4, 99]. However, the approved gene therapy products and clinical trials are still mainly virus-based gene therapy. As the relationship between LNPs or polymeric gene vectors topology and chemical group and the intrinsic mechanism of gene delivery becomes increasingly clear, clinical trials of non-viral vectors are gradually being initiated. Next, this chapter will focus on presenting the current status of non-viral gene therapy in clinical trials.

In 2009, Davis et al. firstly utilized targeted cyclodextrin-containing polymer (CDP) delivery siRNA for patient tumor therapy [100]. This study demonstrated the transition from concept to clinical treatment with non-viral gene therapy. In 2010, Davis et al. reaffirmed the clinical therapeutic potential of non-viral vector-delivered siRNA interference therapy in melanoma patients [101]. In October 2018, the world’s first siRNA drug, Patisiran [102], was approved by the FDA for the treatment of the polyneuropathy of hereditary TTR-mediated amyloidosis using LNP as a vector. Meanwhile, some non-viral vectors are being developed and prepared for pre-clinical trials, For example, Pharma Plc is currently working on developing a non-viral vector-based in vivo gene therapy (AP103) involving HPAEs delivery of *COL7A1* DNA for preclinical trials of RDEB therapy [97]. The Pfizer-BioNTech vaccine BNT162b2 and the Moderna vaccine mRNA-1273 were firstly approved for against SARS-CoV-2 two vaccines. Both vaccines in clinical phase II showed highly protective efficiency (> 94%) [103]. More importantly, both vaccines utilized LNPs that were formulated with ionizable lipid and a modified mRNA sequence encoding the SARS-CoV-2 spike protein with two proline alterations [104]. Currently several mRNA vaccines against infectious viruses using LNP as a vector are proceeding in clinical trials, including Chikungunya virus, Zika Virus and Cytomegalovirus infection [41], according to the Clinicaltrials.gov database. In addition to genetic vaccines against infectious diseases, gene-based cancer vaccines are also widely employed in cancer immunotherapy. The cancer vaccines were used to generate robust T-cell responses against tumor antigens, thereby suppressing or reducing tumor growth. According to data from clinical trials registered on Clinicaltrials.gov, LNP and liposomes are currently being utilized to transport various mRNA for cancer therapy in currently recruiting studies, mainly targeting melanoma, solid tumors, ovarian cancer, urothelial cancer, lymphoma [105, 106], and pancreatic ductal adenocarcinoma [107]. More promisingly, in addition to mRNA immunotherapy trials, many gene therapy clinical trials for cancer are currently being conducted. For instance, PEG-PEI-cholesterol lipopolymer as a gene vehicle deliver the gene encoding IL-12 which has entered clinical phase II. Meanwhile, PEI is used as a vehicle to deliver the DNA plasmid that contains gene regulatory sequences entered clinical phase II. Although gene therapy has demonstrated broad clinical potential, the safety of gene delivery vectors and the high treatment price for genetic rare diseases remains the great challenge to the clinical translation of gene therapy. Therefore, the development of safe and affordable non-viral gene vectors is essential for future development.

## The future development direction of non-viral gene vectors

Based on the promising prospects of gene therapy, we look forward to several major development directions for the high-performance gene vectors in the future. The next-generation vector is expected to include four aspects. (1) Regulating monomer composition and combining with the advantages of various type-polymers, these strategies can alter their hydrophilicity and hydrophobicity, charge density, degradation biocompatibility. (2) Development the different topological structure of polymers, polymers with more precise structures can achieve multi-functional properties including biological targeting, responsive degradation, drug/gene co-delivery, integrated diagnosis and treatment. (3) Functionalized modification for non-viral gene vectors included organ-targeting, light responsiveness, fluorescence visibility, pH or thermal responsive release. (4) Taking inspiration from various sources including the organisms such as cell membrane, cytoplasm and exosome, as well as natural materials such as hyaluronic acid, starch, cellulose, many high-performance gene vectors may be comprehensively designed or developed.

Precision medicine can well overcome individual heterogeneity to achieve safe and efficient gene therapy. Due to the exceedingly broad PDI of polymer, vectors may not be capable of achieving the optimal therapeutic effect for the individual. Combining the advantages of single-chain technology may solve the problem of broad PDI and inaccurate topology. As such, it is necessary to incorporate the data such as individual’s weight, age, physical health during the treatment process. By employing a customized precision nanoparticle, it becomes possible to tailor each individual’s drug dosage to achieve the optimal therapeutic effect.

As most of the current investigation focused on the transfection efficiency of cells, the comprehensive investigation on the transfection mechanism for non-viral gene vectors is still lacking. It is therefore crucial to understand the relationship between the components and structure of vectors and the various stages involved in the transfection process, which can aid in the design of high-performance non-viral gene vectors. Therefore, animal experiments and clinical trials are intensified and can promote the clinical application for non-viral gene vectors. Some promising animal experiments and clinical trials will be implemented in an orderly manner, to ensure the safe and effective use of gene therapy. Furthermore, multidisciplinary design is crucial to the successful development of gene therapy for specific genetic diseases. It requires the integration of various fields, including molecular biology, polymer chemistry, biomedical materials, and others, to design treatment plans and exploit wide-ranging gene delivery systems.

## Data Availability

Not applicable.
